# Long-term standardized forest phenology in Sweden: a climate change indicator

**DOI:** 10.1007/s00484-019-01817-8

**Published:** 2019-10-31

**Authors:** Ola Langvall, Mikaell Ottosson Löfvenius

**Affiliations:** 1 Unit for Field-based Forest Research, Siljansfors Experimental Forest, Swedish University of Agricultural Sciences, Box 74, SE-792 22 Mora, Sweden; 2 Department of Forest Ecology and Management, Swedish University of Agricultural Sciences, SE-901 83 Umeå, Sweden

**Keywords:** Climate change, Plant phenology, Process-based assessments, Phenology models, Boreal forest

## Abstract

Because climate change alters patterns of vegetative growth, long-term phenological measurements and observations can provide important data for analyzing its impact. Phenological assessments are usually made as records of calendar dates when specific phase changes occur. Such assessments have benefits and are used in Citizen Science monitoring. However, these kinds of data often have low statistical precision when describing gradual changes. Frequent monitoring of the phenological traits of forest trees and berries as they undergo gradual change is needed to acquire good temporal resolution of transitions relative to other factors, such as susceptibility to frosts, insects, and fungi, and the use of berries as a food resource. Intensive weekly monitoring of the growth of apical and branch buds and the elongation of shoots and leaves on four tree species, and the abundance of flowers and berries of bilberry and lingonberry, has been performed in Sweden since 2006. Here, we present quantitative methods for interpolating such data, which detail the gradual changes between assessments in order to describe average rates of development and amount of interannual variation. Our analysis has shown the active growth period of trees to differ with latitude. We also observed a change in the timing of the maximum numbers of ripening berries and their successive decline. Data from tree phenology assessments can be used to recommend best forestry practice and to model tree growth, while berry data can be used to estimate when food resources for animals are most available.

## Introduction

One of the most profound effects of a global climate change is how it changes patterns of vegetative growth. Vegetation and soil together provide our principal and most essential needs of food and freshwater. Vegetative growth is highly dependent on a number of factors: a plant’s immediate general environment; its biome, characterized by the site biota; the abiotic conditions such as soil type; water availability; and in particular the local meteorological conditions. Air temperature and humidity, soil temperature and moisture content, wind, solar radiation, and precipitation all exert direct and indirect influences on vegetative growth, survival, and regeneration. Weather conditions obviously vary through time at a particular site, but occasionally meteorological variables can reach extreme values that may be hazardous for some types of vegetation while beneficial to others at different times. Existing vegetation at a site, however, is generally well-adapted to the prevailing meteorological conditions and may, in the long term, cope with most of the natural variations of weather, including periods of unfavorable conditions. The timing of certain weather events may affect the local vegetation, especially seasonally adapted vegetation types. Thus, the type of vegetation found at a particular site tends to reflect the normal weather conditions, and in the long run, the climate.

Climate is generally defined as the average state of the atmosphere and, importantly, the normal range of deviations from it (Dunlop 2001). There needs to be, therefore, a minimum number of samples taken in order to derive a suitably precise estimate of an average value for a variable and its deviation. The period over which samples are taken should ideally also be long enough to include events and extremes that occur more infrequently and should also comprise sequences of several years that include atypical weather conditions. The World Meteorological Organization (WMO) recommendation is to use a time span of 30 years in order to derive “climate normals” and certain 30-year periods (1931–1960, 1961–1990, 1991–2020 etc.) as “reference climate normals”. The main meteorological variables, which are defined and standardized by WMO with long global time series measurements, are air temperature and humidity, atmospheric pressure, wind, and precipitation. In this context, climate relates to the macro- and meso-scale atmospheric conditions, and so to identify any effect of global climate change on vegetation, a corresponding time series and type of phenology are needed.

The boreal forest is a long-lived ecosystem in which individual trees are exposed to various weather events during their lifetime. This generally includes the effects over a 30-year averaging period of weather. Such an ecosystem can therefore provide a source from which long-term measurements and observations can be made that can be analyzed to aid our understanding of the impact of climate change on forest phenology. There are now many studies that demonstrate a shift in the timing of various biological events among both the fauna and flora (e.g. Walter et al. 2002; Brooks et al. 2014; Kolarova et al. 2014; Kullberg et al. 2015), most of which are in agreement with Intergovernmental Panel on Climate Change (IPCC) projections of ongoing climate change (IPCC 2014).

Standard phenological assessments are usually based on the calendar dates when specific changes of phases are seen in nature (Meier et al. 2009). This method has a number of benefits including its easy application to Citizen Science monitoring studies. However, the statistical precision of these kinds of data are often low and of limited use when describing transition phases and gradual changes. In particular, the phenological traits of forest trees and forest berries that change slowly and gradually are easily overlooked. Monitoring these transitions with some precision is needed in order to resolve the exact timing of phase changes and to determine their status with respect to other traits, such as their susceptibility to nocturnal frosts, inimical insects, and fungi, and to determine when berries are available as a food source to various members of the local fauna (Langvall et al. 2001; Hertel et al. 2018). There is currently a need for a quantitative method of monitoring the development of buds and shoots, and of counting berries, in order to evaluate gradual changes and to be able to describe average rates of development and levels of interannual variations. The demand for measurements describing gradual changes of phenological traits has been highlighted in recent phenological research, in which process-based models are produced (Lang et al. 2019), and where ground observations are used to interpret remotely sensed data from PhenoCams (Richardson et al. 2019), drones, or satellite imagery. Furthermore, in a review of current progress and challenges in plant phenology and global climate change, Piao et al. (2019) stated that the lack of process representation in observational phenological records used for producing phenological models induces large uncertainties when predicting phenological responses to future climates.

In the present paper, we present a quantitative method to extend basic phenological observations to include gradual changes in time. The method is then applied, tested, and analyzed using data gathered from four boreal forest sites in Sweden during the period 2006–2017.

## Material and methods

### Assessments

**Fig. 1:**
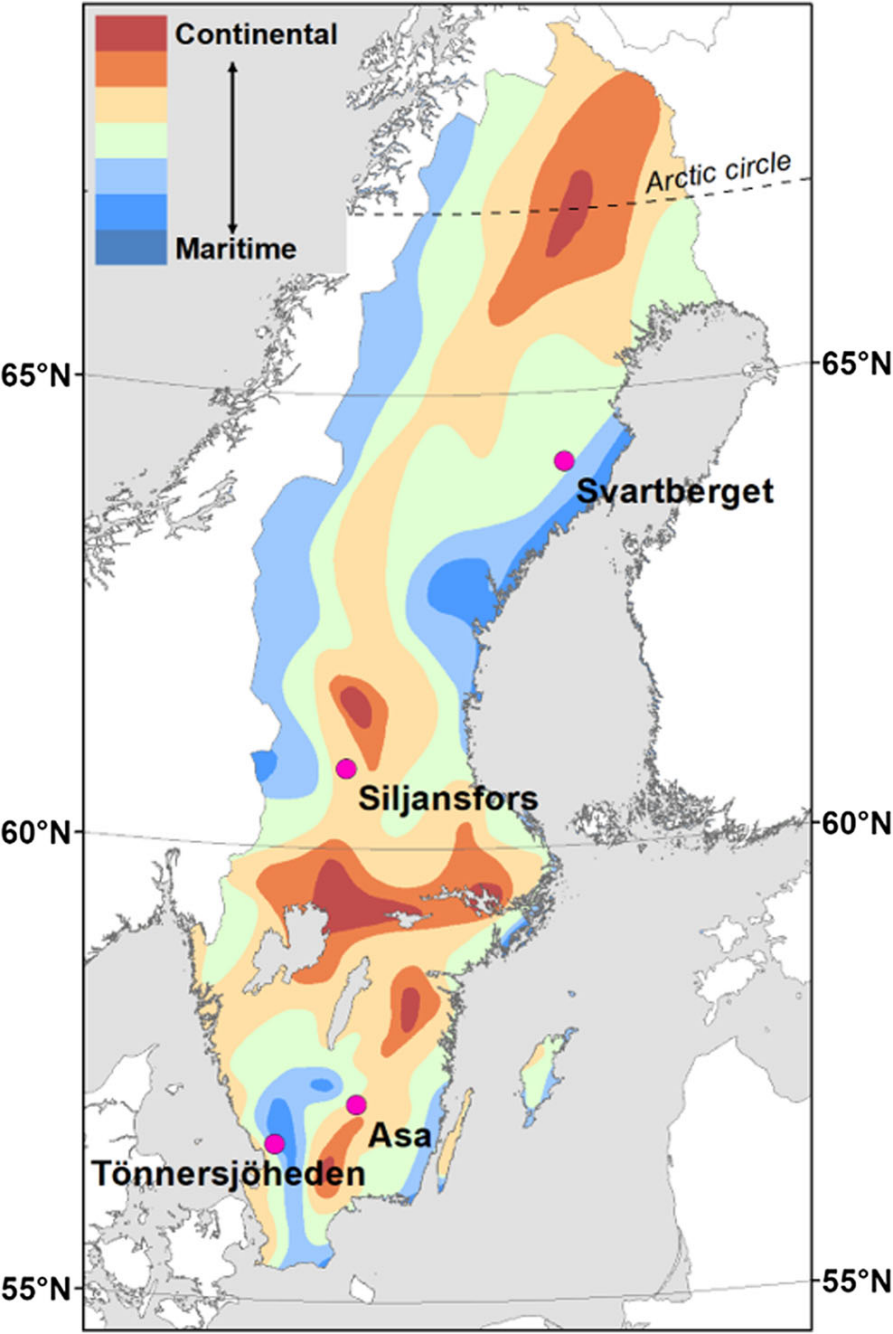
Location of the monitoring sites in relation to latitude and continentality (after Perttu et al. 1978)

Phenological monitoring of forests has been undertaken at four locations in Sweden since 2006. The monitoring sites are located in Experimental Forests permanently staffed and managed by the Faculty of Forest Sciences at the Swedish University of Agricultural Sciences. The sites (Tönnersjöheden, Asa, Siljansfors, and Svartberget) represent a latitudinal gradient from 57° N (Tönnersjöheden and Asa) to 64° N (Svartberget), and a gradient of continentality between Tönnersjöheden, 16 km from the west coast, and Asa, 103 km from the sea (Fig. 1). The Swedish west coast is strongly influenced by the dominant westerly winds, bringing in relatively warm air masses.

Monitoring comprises weekly assessments made from before the beginning of the period of active growth in the spring until the last leaves are shed in the autumn. Assessments are made on the four major tree species in the Swedish forest: Norway spruce (*Picea abies* (L.) Karst.), Scots pine (*Pinus sylvestris* L.), Downy birch (*Betula pubescens* Ehrh.), and Silver birch (*Betula pendula* Roth), as well as on the two dominant wild berries: bilberry (*Vaccinium myrtillus* L.) and lingonberry (*Vaccinium vitis-idaea* L.). Scots pine, Norway spruce, and the two berry species were monitored at all four sites; Downy birch was only monitored at three sites—Asa, Siljansfors, and Svartberget; Silver birch was only monitored at the two southernmost sites—Tönnersjöheden and Asa. We have excluded the Silver birch data from the analyses in this study because any phenological differences due to latitudinal variation could not be compared since the two locations where this species is monitored are at the same latitude.

The assessment methods are adapted to each species individually in order to get the best possible information for the practical use of the data. All tree species are monitored both by recording traditional phenological observations of the dates when different phenological stages are entered, and by continuously measuring (birches and Scots pine) or by assessing a more continuous scale of development phases (Norway spruce) of the development from dormant bud to final size of the elongated shoots or leaves.

For Scots pine, assessments are made on seven young trees at each site, where the apical shoot is easily reached. As the selected trees grow out of reach or have been damaged (e.g. by browsing elk and deer), they are substituted with others. The length of the apical bud is measured with a caliper, and its elongation is recorded until the final length is reached. Additionally, the four traditional stages in the phenology of shoot development are recorded (dormant bud, budburst, elongation reached 2 cm, and final size).

For Norway spruce, assessments are also made on seven young trees at each site. The development and elongation of the apical bud, as well as buds on side branches, are assessed by using the phenology-based Krutzsch index (Krutzsch 1973). The decision to use this index instead of taking actual length measurements was based on the knowledge that assessing the timing of the budburst is crucial, as Norway spruce then enters a period when the new shoot is highly susceptible to frost, and budburst is the most distinctly defined phase in the Krutzsch index. Additionally, as for Scots pine, the four traditional stages in the phenology of shoot development are recorded (dormant bud, budburst, elongation 2 cm, and final size).

**Fig. 2:**
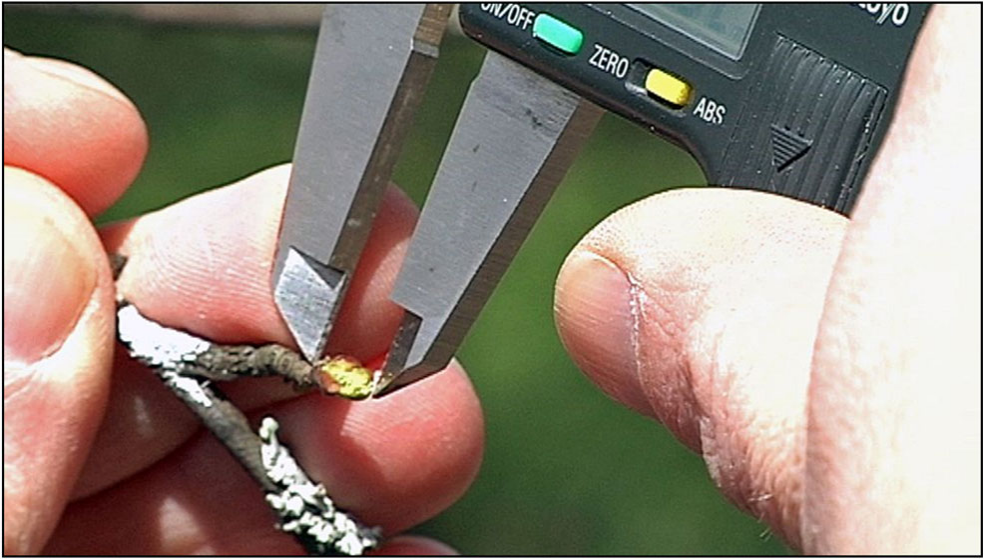
Measuring bud length of Downy birch with a caliper

For the two birch species, observations are made on five young trees of each species, which are also continuously substituted when necessary. Early every spring, seven buds are selected on one of last year’s shoots, on each selected birch tree. Two of these buds serve as substitutes in the event that one or two of the other five should fail to develop to its final size. Each bud, and later on the size of the leaf, is measured with a caliper from the time the bud is still dormant until the leaf has reached full size (Fig. 2). Additionally, nine traditional stages in the phenology of the whole tree are assessed (dormant buds, mouse-ear, leaf-out, final size, autumn colors begin, 50% of autumn colors reached, all leaves in autumn colors, 50% of leaves shed, and all leaves shed).

Berries are monitored in 10 circular sample plots with an area of 0.25 m^2^ and permanently marked out according to a designated pattern such that all plots are separated from each other by at least 10 m. The pattern ensures that the plots are located randomly in the stand with respect to the coverage of the berry shrubs. Within each plot, the abundance of flowers and unripe and ripe berries of the specified berry species is counted at every assessment. Additionally, the coverage of the specific berry species is estimated at the beginning of each season. If a location has less than 5% coverage, that location is abandoned and a new plot is marked out according to the designated pattern.

### Climate data

At each Experimental Forest, the air temperature is measured at standard weather stations as part of a climate monitoring program within the Faculty of Forest Sciences and Unit of Field-based Forest Research. Temperature is measured at screen height (1.7 m) in an open, designated area with shielded and ventilated sensors. Readings are stored in Campbell data-loggers (models 21X, CR10, and CR1000, Campbell Sci., UT, USA) using minute scan to derive daily average temperatures.

### Analysis

The time series of phenological observations and air temperature measurements and their daily average values from the years 2006–2017 were processed and analyzed.

Tree data were processed by first calculating an average of all bud, shoot, and leaf lengths measured on the same day at the same site. For each year, these values where then linearly interpolated between assessment dates, to give a length estimate for every single day during the whole period from dormant bud to full size. On rare occasions, the estimates also included extrapolation if the first assessment was clearly performed after bud dormancy, or if the last assessment clearly had been made before the full size was reached. Since every shoot or leaf had a unique final size every year, the time series of shoot/leaf development was recalculated to a relative scale, i.e., its length was expressed as a percentage of its final size in the same year. Finally, the 12 years of shoot/leaf development time series was averaged for each day of the year, to achieve one “standard” time series for each tree species and site.

The average number of flowers and unripe and ripe berries per square meter was calculated for each assessment day and site using the counts from the 10 sample plots. For each of the three development stages, the numbers of flowers or berries were estimated for each day between assessments, by linear interpolation. As different years yielded different numbers of berries, these interpolated time series datasets for each development stage were rendered into a relative scale with respect to the maximum sum of all flowers and berries counted at one time, for each year and each site separately. The average of the relative numbers of flowers/berries on each day during the season, for the full 12 years, was calculated separately for flowers and unripe and ripe berries, as well as for the total numbers, to achieve a “standard” numeric variation over the season.

The daily average air temperature at each site was progressively summed to calculate the cumulative day-degrees starting at January 1 every year. The threshold value was set to + 5 °C, which means that only that part of the daily average temperature that exceeded + 5 °C was added to the sum, while days with an average temperature below + 5 °C did not add to the sum at all.

The averaged values of bud/shoot/leaf lengths from the assessment dates were then linearly interpolated between the temperature sums achieved at each assessment day. For each year, a time series of bud/shoot/leaf size was related to the cumulative day-degrees during the same year. The average and standard deviation of the yearly time series were then calculated for every day-degree, to achieve a “standard” time series for the relation between bud/shoot/leaf development and cumulative day-degrees and its variation around the mean, for each tree species and site. To achieve the range of day-degrees within one standard deviation of the development of the leaves or shoots, the lower limit was set by adding one standard deviation to the mean and identifying the number of day-degrees at which the particular development level in question (e.g. 50% of shoot length) was reached. The upper limit was set by reducing the mean by one standard deviation and identifying the number of day-degrees at which the development level was reached.

The flower and berry data for the two berry species were also interpolated between assessment date day-degrees, to achieve time series of the number of flowers/berries in relation to the cumulative day-degrees throughout the year. The average and standard deviation of the yearly time series were then calculated at every 5 day-degree step, to achieve a “standard” time series for the relation between the number of flowers and unripe and ripe berries, respectively, and the day-degrees and their variations around the mean, for each berry species and site. This procedure was also repeated for the total numbers of flowers and berries.

Finally, a daily average of the traditional phenological assessments performed on the sample trees was calculated for each species, site, and assessment day. In this study, we used these values to compare results from assessments of phase shifts with the quantitative methods where the development was monitored all the way from dormant bud to full size.

## Results

**Fig. 3:**
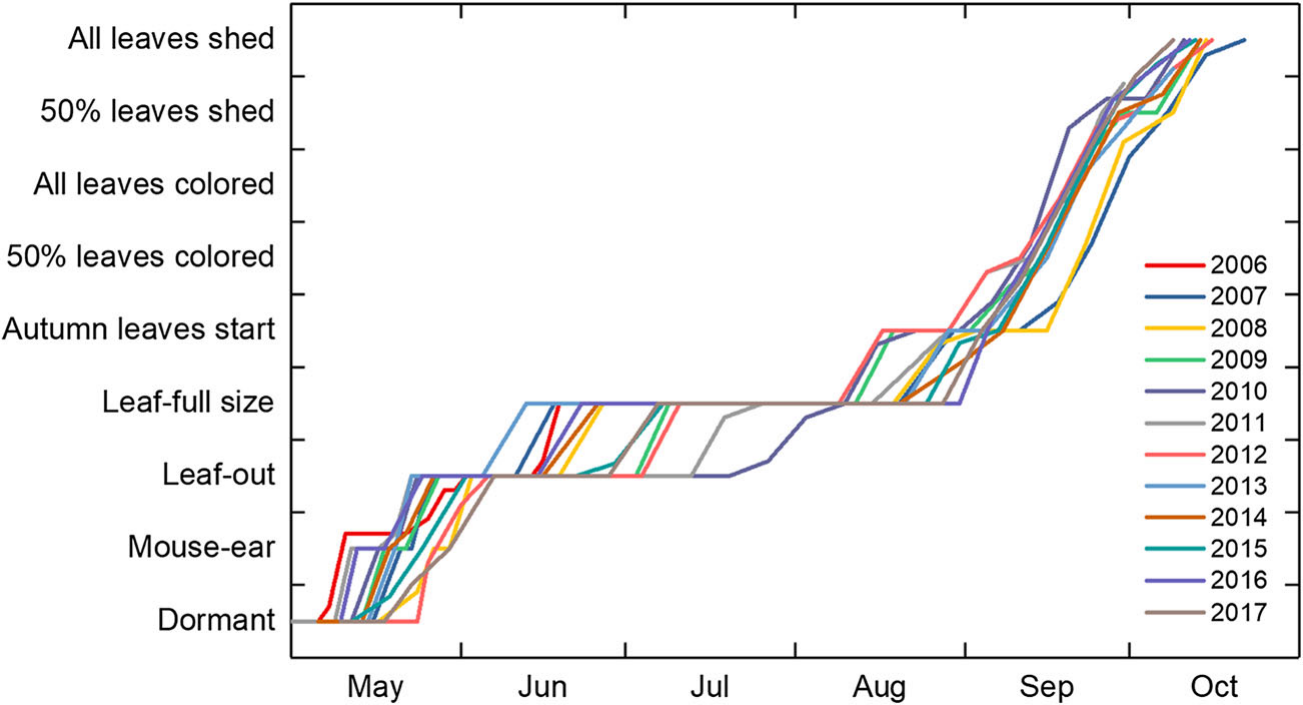
Assessments of traditional phenological phases on Downy birch at Svartberget (lat. 64° N) during the years 2006–2017

The analysis of the traditional phenological assessments made on one site over the 12-year period shows a rather crude scale for the development of the buds and leaves in the spring and summer (Fig. 3). Even if the early stages around budburst seem to be fairly well-defined and accurately observed, the huge differences in time when the leaves were considered to have reached full size reveal a difficulty in the method used to determine the development between the leaf-out and full-size stages.

There is a quite narrow window of time when leaf colors and shedding have started and shift during the autumn of different years, indicating that the initiation of these phases is driven more by day-length than temperature, which drives traits in springtime. The autumn phases cannot be monitored by measurements in the same way as the spring phases but can be assessed with a more or less detailed scale. In our case, we only used three stages for both autumn colors and shedding, viz. the start, 50%, and 100% stages. If assessed at regular and frequent intervals, these stages seem sufficient to define the average rate of development of these traits.

**Fig. 4:**
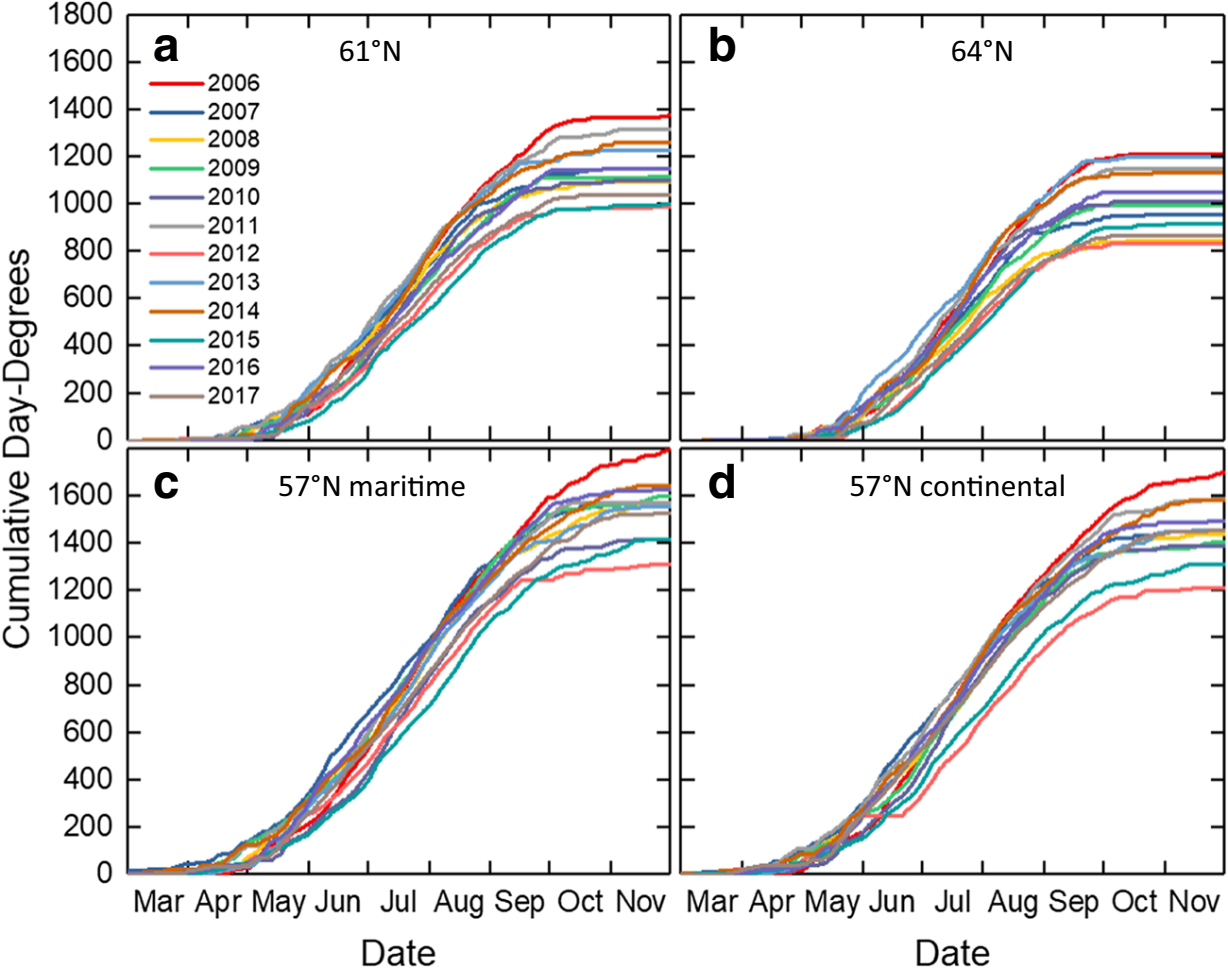
Cumulative day-degrees during the years 2006–2017 at Siljansfors (**a**), Svartberget (**b**), Tönnersjöheden (**c**), and Asa (**d**). Day-degrees were calculated with a threshold value of + 5 °C (see text for details)

Temperature is the main driving factor for active growth in the hemi-boreal and boreal zones in Sweden. The cumulative day-degrees throughout the year is unique for each site and year, but in general, it is lower to the further north where the site is located (Fig. 4). The variation between years by the end of the year was great, around 200 day-degrees in the north and 250 day-degrees in the south during the 12 years. The calendar date when a certain number of day-degrees was reached during the intense growth period, when shoots elongate and leaves are developing into full size, varied by ± 10 days at the Tönnersjöheden, Asa, and Siljansfors sites and by ± 12 days at the Svartberget site.

### Birch

**Fig. 5:**
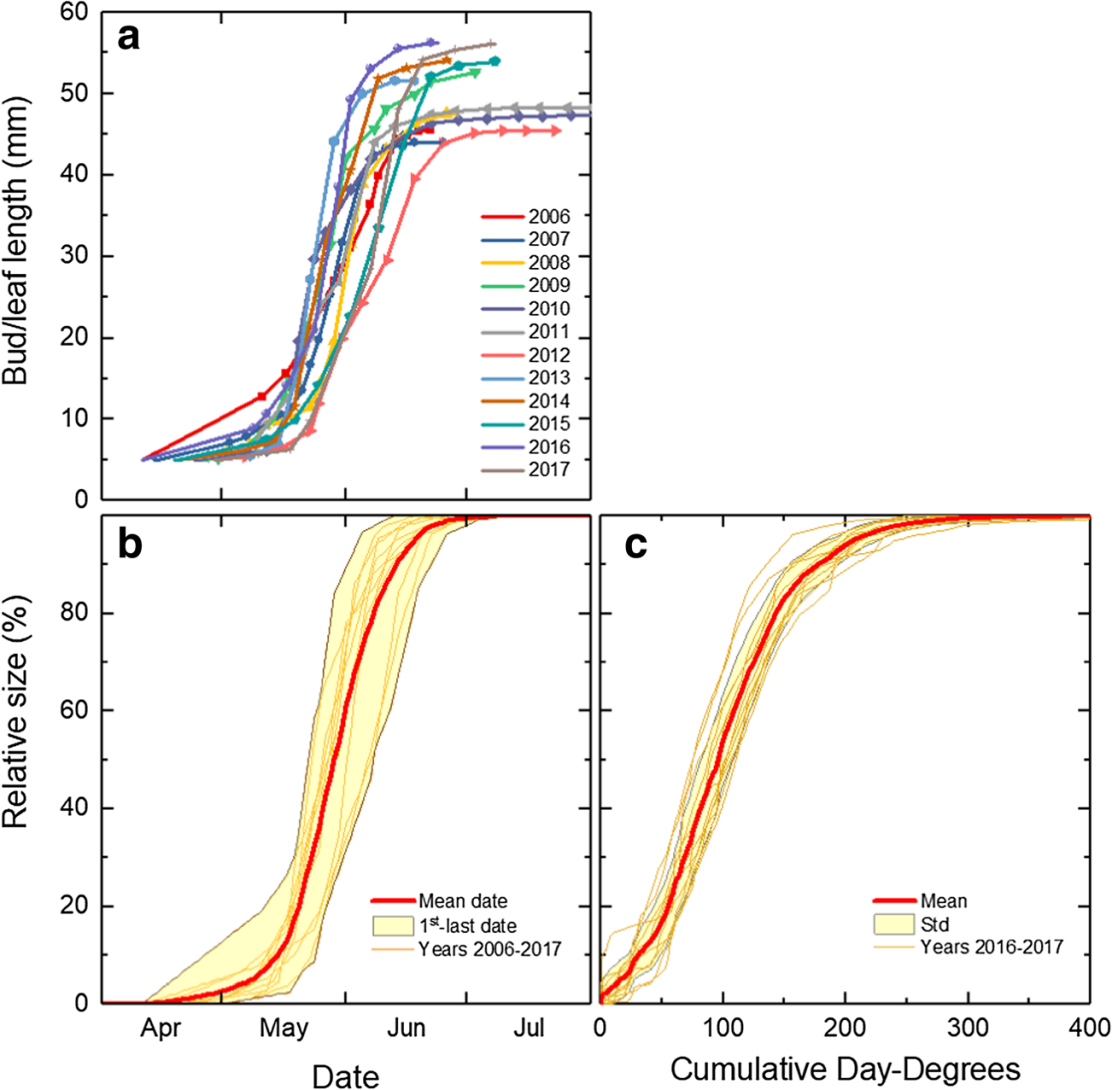
Absolute (**a**) and relative (**b** + **c**) bud and leaf length of Downy birch at Svartberget (lat. 64° N) during the years 2006–2017 vs. date (**a** + **b**) and cumulative day-degrees (**c**). Markers show assessment dates, and lines show interpolations between assessments and extrapolations to dormant bud and final size, respectively. Day-degrees were calculated with a threshold value of + 5 °C (see text for details)

The progress of Downy birch buds and leaves was rather similar from year to year, even though they did not start flushing at the same time and final leaf sizes differed (Fig. 5a). When normalizing the bud/leaf lengths relative to the full final size, the average development from dormant bud to full size over time could be described as a smooth curve with clear boundaries for the minimum and maximum timing of the progression (Fig. 5b). When describing the progression in terms of the relative growth to day-degrees for the actual year, the variation between years narrows down considerably: the standard deviation from mean relative growth was on average 7% during most of the active growth period, i.e., between 10 and 90% of full size, at the Svartberget site (Fig. 5c).

**Fig. 6:**
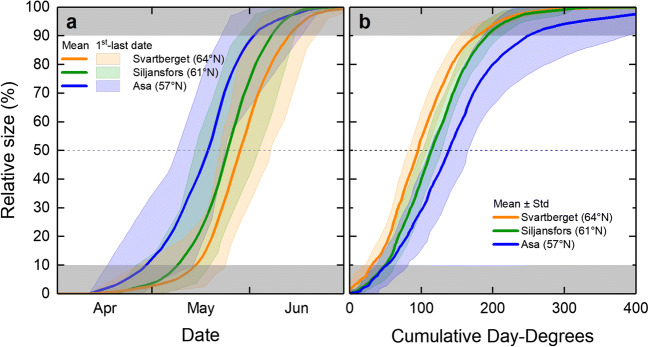
Mean relative bud and leaf size vs. date (**a**) and cumulative day-degrees (**b**) for all three sites with assessments on Downy birch. The shaded areas indicate the period from first to last date (**a**), and standard deviation (**b**), respectively. The gray areas indicate where, in some years, data may have been extrapolated and so have lower accuracy. Day-degrees were calculated with a threshold value of + 5 °C (see text for details)

The average date when the leaves of Downy birch reached 50% of full size occurred as follows: on May 29 at the northernmost site (Svartberget), on May 25 at the mid-Swedish site (Siljansfors), and on May 19 at the southernmost site (Asa) (Fig. 6a).

The average number of day-degrees (range in parentheses) required for Downy birch leaves to reach 50% of full size was 96 (82–108) at Svartberget, 114 (102–128) at Siljansfors, and 138 (111–166) at Asa (Fig. 6b).

### Scots pine

**Fig. 7:**
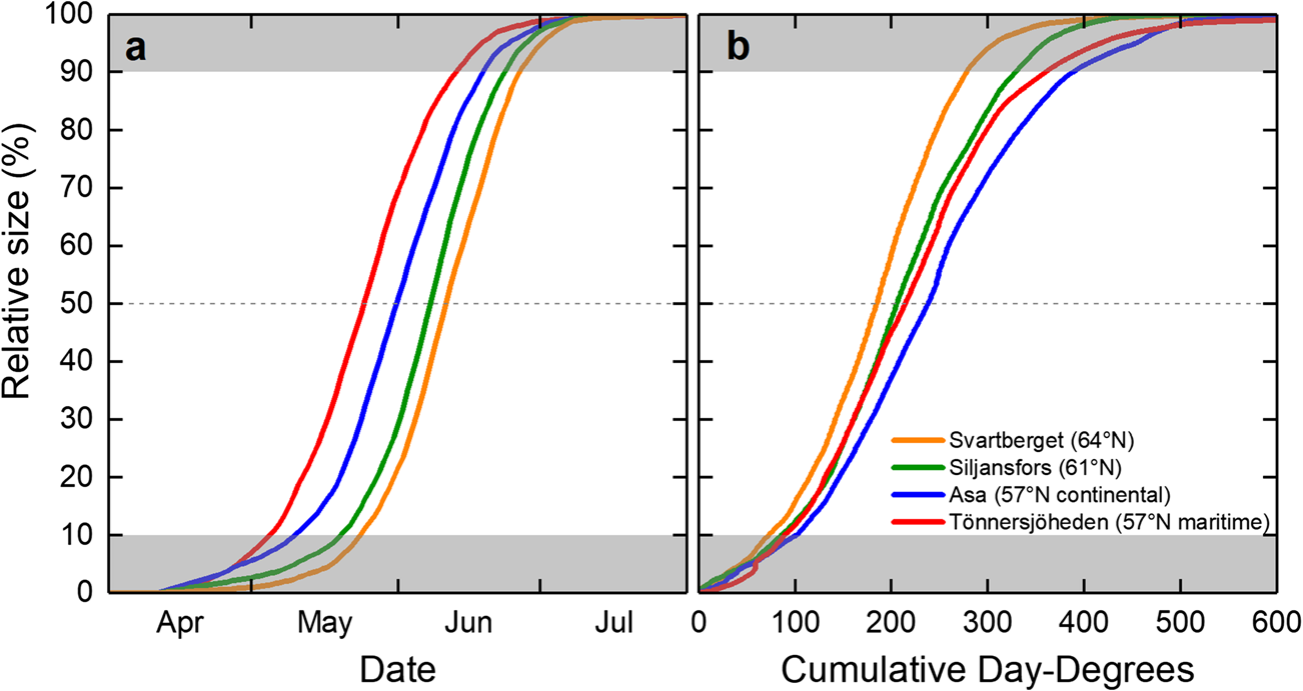
Mean relative bud and shoot length on Scots pine vs. date (**a**) and cumulative day-degrees (**b**) for all sites. The gray areas where, in some years, data may have been indicate extrapolated and so have lower accuracy. Day-degrees were calculated with a threshold value of + 5 °C (see text for details)

The average date when apical shoots of Scots pine reached 50% of full length occurred in June 11 at Svartberget, in June 8 at Siljansfors, in June 1 at the continental site Asa, and in May 25 at the maritime site Tönnersjöheden (Fig. 7a).

The average number of day-degrees (range in parentheses) required for apical shoots of Scots pine to reach 50% of full length was 184 (168–201) at Svartberget, 205 (192–224) at Siljansfors, 214 (191–235) at the maritime site Tönnersjöheden, and 237 (212–264) at the continental site Asa (Fig. 7b).

### Norway spruce

**Fig. 8:**
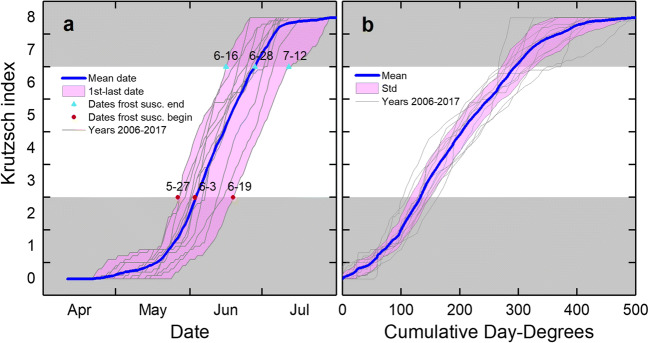
Relative bud and shoot development according to Krutzsch index of Norway spruce at Svartberget (lat. 64° N) during the years 2006–2017 vs. date (**a**) and cumulative day-degrees (**b**). The range from earliest to latest development dates (**a**) and standard deviation from the mean (**b**) are shown as a pink-colored background. The gray areas indicate periods when the buds and shoots are less susceptible to frost. Significant dates (month-day) for identification of the frost-susceptible period are marked in plot **a**. Day-degrees were calculated with a threshold value of + 5 °C (see text for details)

The date when apical buds of Norway spruce reached budburst (Krutzsch index = 3) varied from year to year. At the Svartberget site, the average time range was 23 days, for the 12 years (Fig. 8a). The range at the midpoint and at the end of the frost-susceptible period was even more extended at 27 and 26 days, respectively.

The average number of day-degrees and the range within the standard deviation required for apical buds of Norway spruce to reach budburst was 133 (115–150) at Svartberget (Fig. 8b). To reach the midpoint and the end of the frost-susceptible period, 203 (182–224) and 294 (271–328) day-degrees are required, respectively. The standard deviation on the Krutzsch index scale at the number of day-degrees when the average budburst occurred was 0.55 index step in the Krutzsch scale, and slightly more, 0.66, at the number of day-degrees when the average shoot development reached the stage where frost susceptibility decreased (Krutzsch index = 7).

**Fig. 9:**
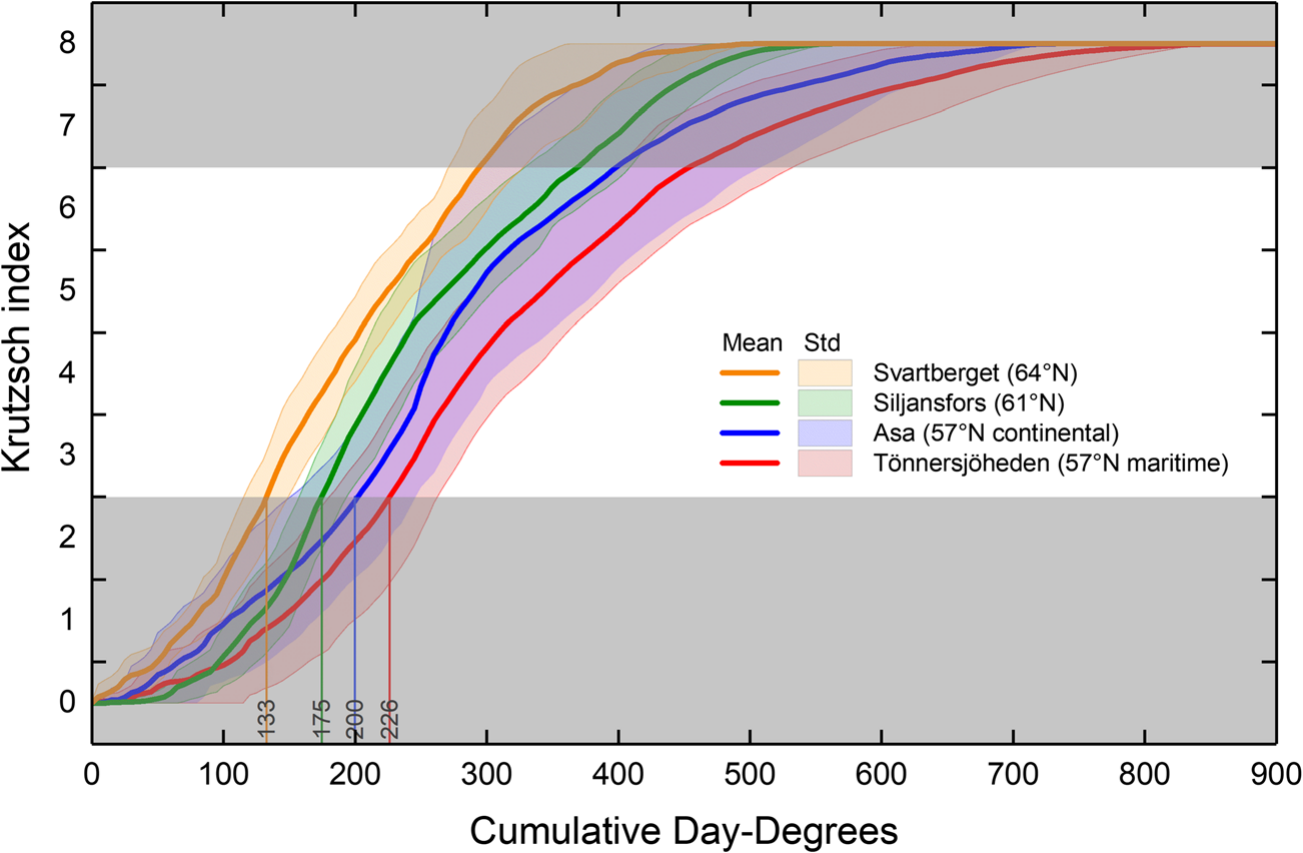
Mean relative bud and shoot development of Norway spruce according to Krutzsch index at all sites. Drop-down lines show when shoots reach Krutzsch index 3 (budbreak). Standard deviation is shown as colored background. The gray areas indicate periods when the buds and shoots are less susceptible to frost. Day-degrees were calculated with a threshold value of + 5 °C (see text for details)

The average number of day-degrees (range in parentheses) required for apical buds of Norway spruce to reach budburst was 133 (115–150), 175 (158–194), 200 (146–246), and 226 (181–263) at Svartberget, Siljansfors, Asa, and Tönnersjöheden, respectively (Fig. 9). Since the accumulation of day-degrees varies between sites, the different average calendar dates for budburst, which were almost the same for the two southernmost sites, May 26 and May 27 at Tönnersjöheden and Asa, respectively, and for the two northern sites, June 2 and June 3 at Siljansfors and Svartberget, respectively (data not shown) it is worth noting.

### Bilberries and lingonberries

**Fig. 10:**
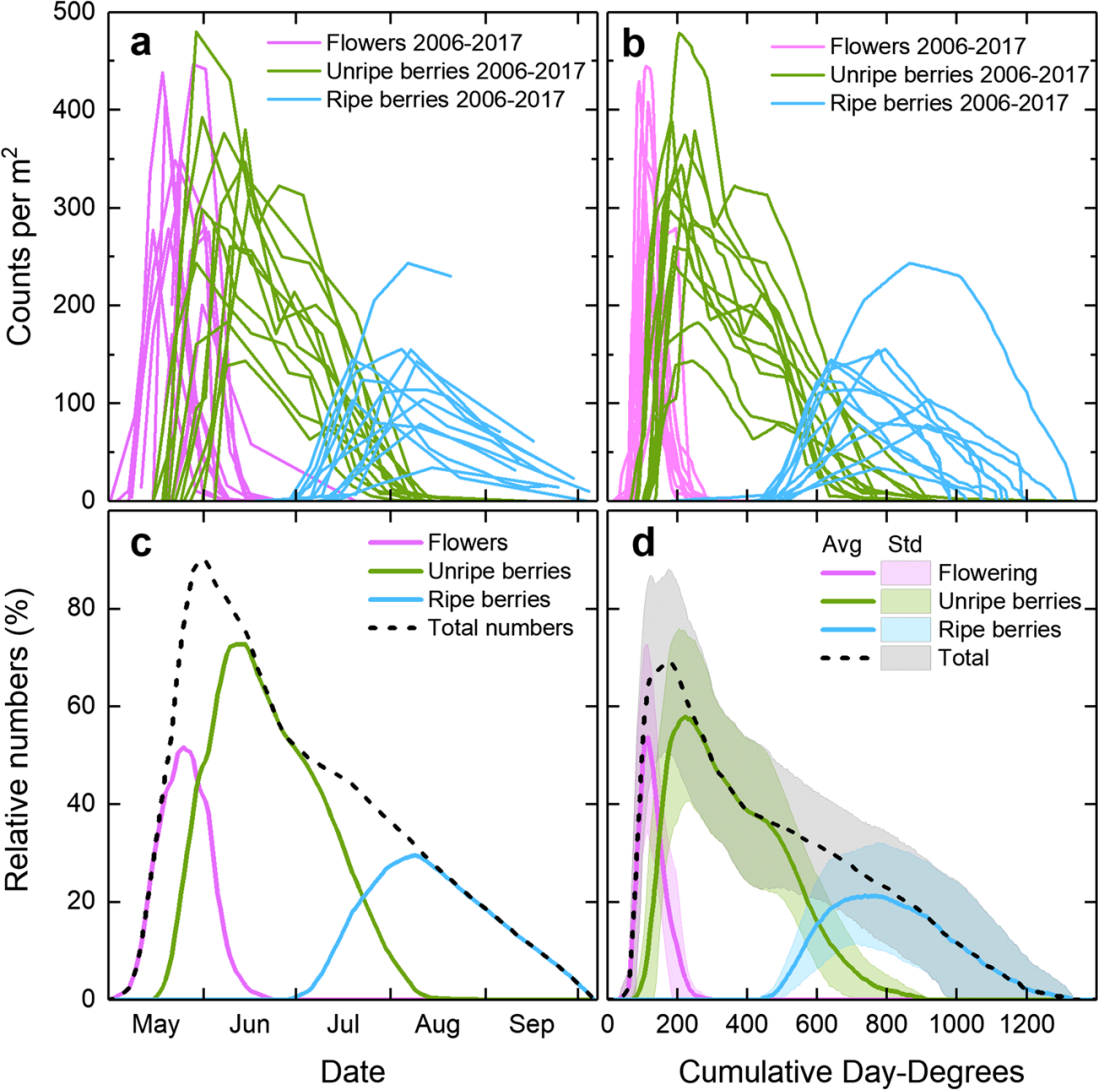
Absolute (**a** + **b**) and relative (**c** + **d**) numbers of flowers and unripe and ripe bilberries at Siljansfors (61° N) for single years (**a** + **b**) and the mean for all years 2006–2017 (**c** + **d**) vs. date (**a** + **c**) and cumulative day-degrees (**b** + **d**). Standard deviation is shown as colored background in **d**. Day-degrees were calculated with a threshold value of + 5 °C (see text for details)

The assessments of bilberries revealed large annual variations in the timing of flowering, the date when unripe and ripe berries occurred, and in the actual numbers of flowers and unripe and ripe berries found on the plots at the Siljansfors site (Fig. 10a). When the actual numbers were transformed into the relative numbers with respect to the total, and averaged for all 12 years, a distinct pattern emerged: there was a narrow period of flowering which gradually transferred to a long period of unripe berries and, again, the gradually ripening berries could be distinguished (Fig. 10c). On average, flowering peaked on May 26. The bilberries ripened during a rather long period, from July 4 until August 11, and on average reached the maximum number of ripe berries in August 9. Interestingly, the number of ripe berries was on average, only one third of the maximum total numbers found on the plots. In addition, the ripe berries disappeared gradually during a rather short period at the Siljansfors site, from mid-August until the end of September.

When the timing of flowering was analyzed relative to day-degrees at the Siljansfors site, the flowering period was seen to narrow to a rather short and well-defined period between 70 and 200 day-degrees, with a peak at 115 day-degrees (Fig. 10d). Similarly, unripe berries were present between 115 and 650 day-degrees, peaking at 220 day-degrees; ripe berries were present between 555 and 1030 day-degrees, on average, peaking at 720 day-degrees. The variations around the means of unripe and ripe berries were greater than for flowering.

**Fig. 11:**
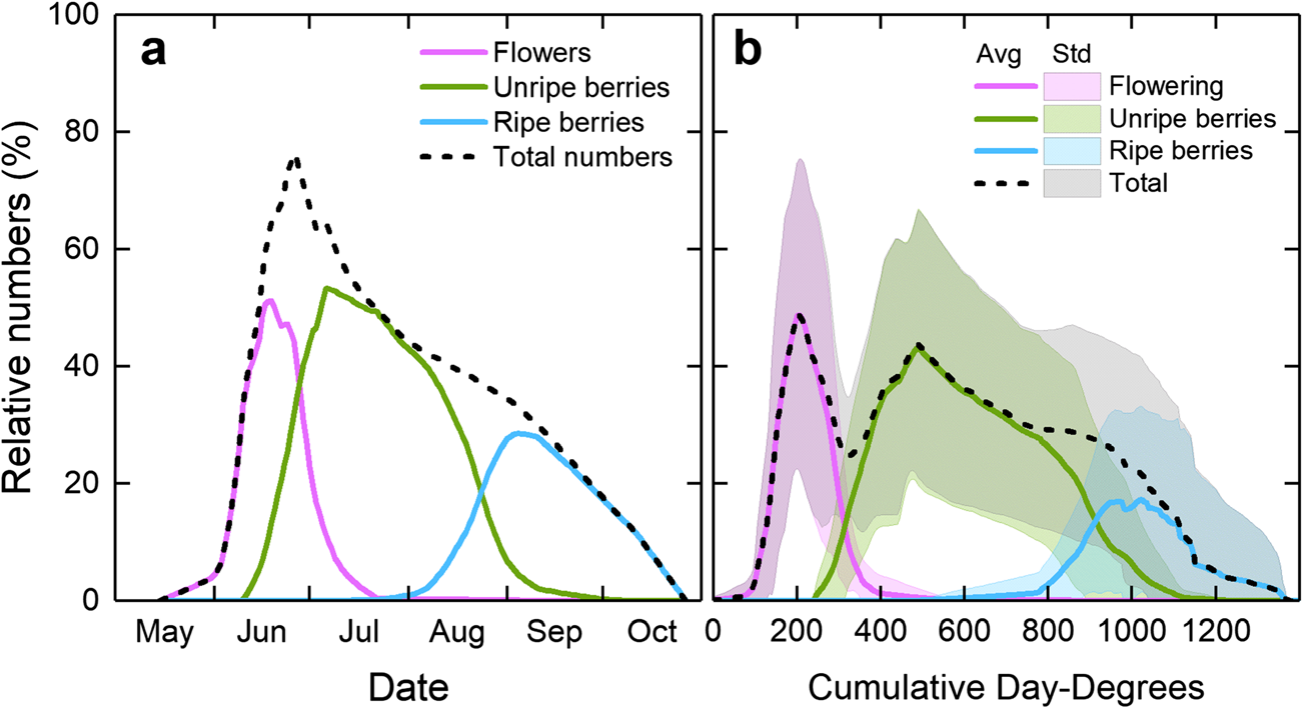
Mean relative numbers of flowers and unripe and ripe lingonberries at Siljansfors (61° N) for all years 2006–2017 vs. date (**a**) and cumulative day-degrees (**b**). Standard deviation is shown as colored background in **b**. Day-degrees were calculated with a threshold value of + 5 °C (see text for details)

The same pattern of annual variation in the timing of flowering, and when unripe berries and ripe berries occurred, and on the actual numbers of flowers, and of unripe and ripe berries that were present on the plots at Siljansfors, was also apparent for lingonberry (data not shown). The relative numbers of lingonberries also showed a distinct pattern, with a rather narrow period of flowering that gradually transferred to a long period of unripe berries that gradually ripened (Fig. 11a). Flowering peaked on average on June 19. The lingonberries ripened from August 1 until September 21, reaching the maximum number of ripe berries in September 4, on average. The number of ripe berries was on average 50% of the maximum total numbers found on the plots. As with the bilberries, the ripe lingonberries at Siljansfors disappeared gradually over a rather short period between the beginning of September and the end of October.

On average, flowering occurred between 120 and 320 day-degrees, peaking at 205 day-degrees (Fig. 11b); unripe berries were present between 305 and 940 day-degrees, peaking at 490 day-degrees; ripe berries were found between 715 and 1130 day-degrees, with a peak at 1025 day-degrees.

## Discussion

The phenology of forest vegetation, represented here by below-canopy berries and long-lived standing trees, takes places over a period that includes both short-term weather effects as well as long-term gradual changes related to the climatic time scale. To evaluate the effect of climate change on forest vegetation, phenological observations need to be long term and the observational methodology robust and independent of the observers. By normalizing and interpolating observations, gradual changes can be analyzed and differences between sites revealed. Although the time period presented here (2006–2017) is insufficient in the context of climate change, an intriguing pattern of continental and latitudinal influences is clearly demonstrated. Indeed, all tree species required fewer day-degrees at higher latitudes to reach 50% of their final elongation, although this may include an effect of increasing day length and a shorter vegetation period with latitude. Differences can be explained by the genetic adaptation of each species to the local environment at the different sites, which in turn gives rise to different provenances within the species. However, such adaptation will not be able to respond fast enough to match the speed of anthropogenically induced climate change.

In the present study, we have shown that a quantitative method of assessing phenological traits, which are actually gradual transformations from one stage to another (e.g. from dormant bud to fully elongated shoot), can easily be used to describe the succession. Moreover, since the measured values can be interpolated and plotted on a relative scale, the developmental process can be analyzed in more detail than has hitherto been possible with traditional phenological observations of one or a few steps usually used to define the process. These steps, in general, cannot be assessed with the same accuracy, simply because it is not possible to predict the timing of future states: for example, it is not possible to assess a 50% elongation state if the final size is unknown at the time when a 50% elongation assessment needs to be made.

An important drawback of our method is the need for trained personnel who are able to make the necessary intensive observations at the required frequency, as well as endure the rigorous field conditions, especially if data are required to generalize phenology over a large geographical area. Our method may therefore be limited to implementation by a few professional research stations or similar sites, which are permanently staffed in near suitable forested areas. However, geographical limitations might be overcome if our quantitative method could be used at a few sites, together with traditional phenology assessments made at the same site, and combined with data acquired through Citizen Science initiatives. This would increase the value of Citizen Science data by enhancing its precision through its correlation with professionally acquired data.

Our interpolation method loses accuracy to some extent if, during a particular year, buds start to elongate before it has been possible to get into the field to take any measurements. In such cases, the estimated starting point has less precision. Another source of error can be a distorted relative scale. This can arise if the final size of a bud, leaf, or shoot is not determined definitively by two successive measurements being identical. In our analyses, we used linear interpolation between measurements. This is adequate when data cover short time periods, but over longer periods, the interpolation might improve if other functions are used, e.g. exponential or logarithmic, at particular developmental stages. The whole time series could also be evaluated by regression analysis, to fit a model to the assessment values, e.g. a Weibull function. However, the advantage with our current interpolation method is that we get daily values or, in the case where we use cumulative day-degrees as the driving variable, a value attributable to each day-degree of every specific year. The calculation of an average development of the trait over several years is then much simplified. Some traits which are important to define, such as budburst on Norway spruce, are not well-defined by the quantitative method, because relative size does not reveal when a specific bud actually burst or when needles are suddenly exposed to nocturnal frosts. In such cases, either the assessments to be based on a relative scale, such as the Krutzsch index, or the assessments to be supplemented with observations of the specific phase shift are needed.

The interpolation method, as we describe it here, seems to work very well when describing phenological traits that develop over a rather long season of vegetative growth, which have a strong correlation with air temperatures that are driven by the kind of seasonality we experience in boreal latitudes. In latitudes where seasonality is less pronounced, and for species that do not have to adapt to cold winters, such as evergreen broadleaf species, we need to develop specific models, in order to describe the quantitative factors that drive their phenology, e.g. light intensity, high temperatures, and precipitation.

The results from the quantitative method presented here can be a useful research tool in building process-based models to describe growth patterns in which temperature is the forcing factor. Another example might be where models are used to estimate seasonal variations in the flux of greenhouse gases into and out of the forest as flushing patterns and leaf area change. With longer time series, trends driven by climate change should be easier to identify by comparing average development times over shorter periods.

The use of process-based models has been used in practical forestry and could be used more widely to predict those periods when trees are susceptible to environmental factors such as nocturnal frosts (Langvall 2000; Hannerz 1999), or are most susceptible to fungal diseases (e.g. *Melampsora populnea* (Pers.) P.Karst. and *Gremmeniella abietina* (Lagerb.) M.Morelet) or insect attack (e.g. *Operophtera brumata* (Linnaeus, 1758) and *Diprion pini* (Linnaeus, 1758)). This is especially useful when producing scenarios of the impact of climate change and when planning how to adapt to these effects (Langvall 2011).

Estimates of the yearly production of bilberry and lingonberry are published in the official national forest statistics (Nilsson et al. 2017). Research, such as our present study, that can generate predictions of berry abundance and ripening could have implications for predicting the reproductive success of the brown bear and their behavior (Hertel et al. 2018, 2019). Moreover, the general public have benefited from forecasts of the abundance and ripening of forest bilberries and lingonberries published in mass media by the Swedish University of Agricultural Sciences.

In the present study, we have quantified the loss of berries below their maximum potential productivity calculated from the numbers of flowers and unripe berries present earlier in the season and derived results, at a higher resolution than those reported by Laakso et al. (1990) and Boulanger-Lapointe et al. (2017). Because we recorded the timing of flowering and ripening, it is possible to investigate further the state of key environmental conditions and the timing of other events and factors that lead to the successful development of flowers to ripe fruits—e.g. conditions for pollinators (Eckerter et al. 2019)—during those periods that affect the abundance of ripe berries at the end of the season. The continuous reduction of ripe berries in the autumn could also be correlated to the effects of environmental factors in the same manner.

## Conclusions

By using intensive monitoring and the interpolation and normalization of data, we have shown that the dynamics of phenological phase transitions can be analyzed at high resolution, especially for traits that change gradually over a relatively long period such as shoot development.

Tree phenology assessments can be used to recommend certain forestry practices and to parameterize models of tree growth, while berry data can be used to estimate the availability of food resources for wild animals.

Long-term standardized forest phenology observations provide appropriate data for climate change studies. The trees in a boreal forest withstand decades of weather exposure, possibly even centuries, and the large area covered by boreal and hemi-boreal forest in the northern hemisphere provides an environment with ideal temporal and spatial scales relevant and well-matched to the scales of global climate change.
